# Tuberculosis- and Diabetes Mellitus-Associated Knowledge, Stigma, and Health-Seeking Behaviour in Ghana

**DOI:** 10.3390/ijerph23081050

**Published:** 2026-08-13

**Authors:** Edmond Kwaku Ocloo, Adwoa Asante-Poku, Ishaque Mintah Siam, Nii Arku Laryea, Emmanuel Frimpong Gyekye, Emmanuel Afreh Kyei, Stephen Osei-Wusu, Michelle Yeboah-Manu, Eric Koka, Richard Adjei Akuffo, Daniel Okyere, Prince Asare, Augustine Asare Boadu, Emelia Konadu Danso, Phillip Tetteh, Kenneth Mawuta Hayibor, Theophilus Afum, Isaac Darko Otchere, Nelly Arthur, Sylvester Owusu Amoako, Yaw Adusi-Poku, Charlotte-Alberta Cato, Abraham Adjei, Jane Afriyie-Mensah, Dorothy Yeboah-Manu

**Affiliations:** 1 Department of Bacteriology, Noguchi Memorial Institute for Medical Research, University of Ghana, Legon, Accra P.O. Box LG 581, Ghana; isiam@noguchi.ug.edu.gh (I.M.S.); eakyei@noguchi.ug.edu.gh (E.A.K.); sosei-wusu@noguchi.ug.edu.gh (S.O.-W.); dokyere@noguchi.ug.edu.gh (D.O.); pasare@noguchi.ug.edu.gh (P.A.); aboadu@noguchi.ug.edu.gh (A.A.B.); edanso@noguchi.ug.edu.gh (E.K.D.); ptetteh@noguchi.ug.edu.gh (P.T.); kenhayibor@gmail.com (K.M.H.); theophilusafum@gmail.com (T.A.); iotchere@noguchi.ug.edu.gh (I.D.O.); dyeboah-manu@noguchi.ug.edu.gh (D.Y.-M.); 2 Department of Sociology and Anthropology, University of Cape Coast, Cape Coast PM, Ghana; ekoka@ucc.edu.gh; 3 Department of Epidemiology, Noguchi Memorial Institute for Medical Research, University of Ghana, Legon, Accra P.O. Box LG 581, Ghana; nlaryea@noguchi.ug.edu.gh (N.A.L.); rakuffo@noguchi.ug.edu.gh (R.A.A.); 4 Department of Virology, Noguchi Memorial Institute for Medical Research, University of Ghana, Legon, Accra P.O. Box LG 581, Ghana; kwafrimpong@gmail.com; 5 University of Ghana Medical Centre, Legon, Accra P.O. Box LG 25, Ghana; yeboahmanumichelle@gmail.com; 6 Department of Chest Diseases, Korle-Bu Teaching Hospital, Accra P.O. Box 77, Ghana; nellyarthur6776@gmail.com (N.A.); skoamoako@gmail.com (S.O.A.); adjeiabraham285@gmail.com (A.A.); jafriyie-mensah@ug.edu.gh (J.A.-M.); 7 National Tuberculosis Control Programme, Ghana Health Service, Accra P.O. Box KB 493, Ghana; adusipokuyaw@gmail.com; 8 Mamprobi Hospital, Mamprobi P.O. Box MP 772, Ghana; baabacato@yahoo.com

**Keywords:** tuberculosis, diabetes mellitus, knowledge, stigma, health-seeking behaviour

## Abstract

**Highlights:**

**Public health relevance—How does this work relate to a public health issue?**

- Tuberculosis and diabetes mellitus are significant public health concerns worldwide.
- Tuberculosis negatively affects an individual’s glucose tolerance, thereby increasing the risk of developing diabetes mellitus.

**Public health significance—Why is this work of significance to public health?**

- By employing a mixed-methods research approach involving a cross-sectional survey and in-depth interviews, we explored community knowledge, stigma, and health-seeking behaviour related to tuberculosis and diabetes mellitus.
- Our findings suggest that addressing cultural barriers, stigma, and financial costs associated with TB and diabetes care may help increase patients’ penchant for biomedical treatment.

**Public health implications—What are the key implications or messages for practitioners, policy makers and/or researchers in public health?**

- Our study reveals that social determinants of health, such as poverty, local beliefs, stigma, and poor health personnel attitudes, may influence TB patients’ preference for traditional remedies over biomedical treatment.
- This study suggests that interventions toward improving physician–patient relationships may increase patients’ interest in biomedical care, as healthcare providers’ unwelcoming attitudes toward patients were highlighted as a significant impediment to seeking care from health facilities.

**Abstract:**

Introduction: Tuberculosis [TB] and diabetes mellitus (DM) remain major public health concerns worldwide. TB negatively affects an individual’s glucose tolerance, increasing the risk of developing DM. Similarly, DM reduces the host’s ability to fight TB infection and increases the likelihood of developing TB and poor response to TB medication. This study analysed stigma and health-seeking behaviour in a sub-district of Accra Metropolis. Methods: We used a mixed-methods research approach involving a cross-sectional survey and in-depth interviews to explore community knowledge, stigma, and health-seeking behaviour related to TB and DM. A total of 437 individuals were selected for a survey using a simple random sampling technique, while 22 participants, including three TB patients, four nurses, one medical doctor, four students from a nursing and midwifery school, five students from a school of hygiene, and five community members, were involved in the study. Healthcare providers and patients were purposively selected, while students and community members were recruited through convenience sampling. Descriptive and logistic regression analyses were performed on the quantitative data using STATA, while the qualitative data were thematically analysed using the MAXQDA software. Results: Participants’ age, sex, and educational levels were significantly associated with their knowledge of TB and DM. Males were 0.600 times as likely as females to be knowledgeable about DM (95% CI: 0.386–0.934, *p* = 0.024). Social determinants of health, such as poverty, local beliefs, stigma, and negative attitudes from healthcare workers, may influence TB patients’ preference for traditional remedies over biomedical treatment. Conclusion: Therefore, we observed that addressing cultural barriers, stigma, and financial costs related to TB and diabetes care will help increase patients’ willingness for biomedical treatment.

## 1. Introduction

Tuberculosis (TB) and diabetes mellitus (DM) are significant public health concerns worldwide. TB is the leading cause of death globally from a single infectious agent, resulting in nearly twice as many deaths as HIV/AIDS [1]. In 2023, TB caused the deaths of at least 1.25 million people [2]. Statistics indicate that the WHO African region accounts for a quarter of global TB cases and, along with Southeast Asia, reported 85% of all TB deaths worldwide that year [3]. Diabetes mellitus, commonly referred to as diabetes, is a rapidly growing chronic disease in the 21st century, leading to disability and premature death among individuals in their most productive age group [4]. DM has reached pandemic levels, with a prevalence rate of 10.5%, resulting in 6.7 million deaths globally in 2021 [4]. Coincidentally, diabetes is also mostly prevalent in lower- and middle-income countries with high morbidity rates and, regrettably, patients there tend to die earlier than their counterparts in developed countries [5]. DM cases worldwide increased from 151 million in 2000 to 589 million in 2024 among adults aged 20–79 [5]. In 2024, nearly 317,400 people in Ghana were diagnosed with DM, reflecting a 13% increase from the 2019 figure of 281,100 [5].

TB and DM have a bidirectional relationship. TB negatively impacts an individual’s glucose tolerance and the management of DM [6,7,8]. On the other hand, diabetes increases the risk of developing TB by two to threefold and may lead to negative treatment outcomes for TB [9]. Estimates of TB-DM comorbidity in Ghana remain limited, although Asante-Poku and colleagues found that the prevalence of DM in TB patients is twice that of the general population [6]. Diabetes has been recognised as the fifth highest risk factor for TB, and current evidence suggests that if the rise in diabetes continues unabated, we may struggle to achieve the global reduction targets for TB incidence (80%) and mortality rates (90%) by 2030 [10].

Financial constraints, inadequate knowledge, erroneous beliefs, and misconceptions about causes, modes of transmission, symptoms, treatment, and stigma not only affect the healthcare-seeking decisions of TB and DM patients [11,12,13,14,15,16] but also foster a preference for alternative care [spiritual, faith, and traditional healing] over biomedical care [17,18]. This results in poor treatment adherence, delays in diagnosis, and an extensive spread of TB among healthy community members [19]. The horror of social isolation, prejudice, stigmatisation, and discrimination by family members, healthcare staff, and community members may hinder TB and DM prevention and control efforts, culminating in treatment default, concealment of health status, and delays in healthcare-seeking and diagnosis by patients [20]. However, studies appear limited on how social interpretations or understandings of TB and DM influence patients’ healthcare-seeking decisions in Ghana. Therefore, we explored community knowledge, attitudes, stigma, and health-seeking behaviour associated with TB and DM in Ghana, which may form the basis for developing people-centred social interventions to improve patients’ quality of life.

## 2. Materials and Methods

### 2.1. Study Setting

This study was conducted in the Ablekuma South Sub-Metropolitan District of Ghana from January to February, 2025. The Ablekuma South Sub-Metropolitan District is a sub-metro within the Accra Metropolitan Assembly (AMA) with a population of 307,301 [21]. It shares boundaries with the Ablekuma Central Municipal Assembly, Ablekuma North Municipal Assembly, and Ashiedu Keteke Sub-Metropolitan District Council. The Metropolitan Assembly is home to Ghana’s oldest and largest teaching hospital, the Korle-Bu Teaching Hospital.

### 2.2. Research Design

We adopted a mixed-methods research design, involving a cross-sectional survey and in-depth interviews with individuals aged 18 and older to assess their knowledge, attitudes, stigma, and health-seeking decisions related to TB and DM. Using Daniel’s (1999) formula, *n* = *Z*^2^ x *P* x (1 − *P*)/*d*^2^, a sample size of 384 was derived [22]. However, to address non-responses and missing information, the sample size was increased to 437. A total of 437 individuals were surveyed using a simple random sampling technique. A “yes” or “no” was written on paper and tossed for potential participants to draw. People who drew a “yes” from the paper were included in the study, provided they were willing to participate. A validated questionnaire was administered to the participants. For the qualitative interviews, 22 individuals [3 TB patients; 4 nurses and 1 medical doctor at the Chest Clinic of the Korle-Bu Teaching Hospital caring for TB patients; 4 students of the Korle-Bu Nursing and Midwifery School; 5 students of the Accra School of Hygiene; and 5 community members] were purposively recruited to provide deeper insights into the two diseases and complement the quantitative data. The interviews were conducted using an interview guide and were tape-recorded.

### 2.3. Data Analysis

Quantitative data were cleaned in Microsoft Excel 2019 (Microsoft Corporation, Redmond, Washington), checked for completeness, and imported into Stata version 17 for statistical analysis. Descriptive statistics summarised participant characteristics and were presented in tables with proportions. Inferential analysis of responses to knowledge and stigma questions about TB and DM was coded, with correct responses as “1” and incorrect responses as “0”. Composite scores for knowledge and stigma toward TB and DM were calculated separately. Depending on the distribution of each composite score, participants were categorised as having poor knowledge or stigma (score below the median) or good knowledge or stigma (score at or above the median). This process resulted in four binary dependent variables: TB knowledge, DM knowledge, TB stigma, and DM stigma. Binary and multivariable logistic regressions were used to assess associations between participant characteristics and each of the four outcomes. Statistical significance was set at a *p*-value ≤ 0.05. Model fit was assessed using the Hosmer–Lemeshow goodness-of-fit test, with all *p*-values > 0.05. Multicollinearity among predictors was assessed using variance inflation factors (VIFs), all <5. Qualitative interviews were analysed thematically using the MAXQDA software version 26. The analysis proceeded with (i) listening to the audio recording of each interview, (ii) reading the field notes taken during the interview, and (iii) reading the original transcripts and translations to validate consistency across data sources and ensure familiarity with the data before identifying themes and creating a codebook. This approach ensured the identification and successive validation of a priori themes initially developed by the lead author. Two authors [E.K.O. and E.G.F.] coded the data, and a third person [N.A.L.] authenticated the various themes and codes that emerged. Key themes were identified, and matrices were developed to discern patterns and relationships among the themes. All datasets are kept in a secure institutional REDCap of the Noguchi Memorial Institute for Medical Research, University of Ghana.

This study received ethical approvals from the Noguchi Memorial Institute for Medical Research Review Board (Figure S1) and Ghana Health Service Review Committee (Figure S2).

## 3. Results

### 3.1. Sociodemographic Profiles of Participants

Out of the 437 participants, 284 (65.0%) were females, and 153 (35.0%) were males (Table 1). The participants represented a wide age range, with the largest percentage (55.6%) in the 18–35 age group, followed by those aged 36–59 (33.0%). Most had elementary education (42.1%), secondary education (24.5%), or tertiary education (22.2%); 11.2% had no formal education. In terms of occupation, the largest group was self-employed individuals (58.4%), followed by students (22.7%) and the unemployed (14.6%). In terms of religious affiliation, the majority identified as Christians (85.1%), followed by Muslims (11.0%), African Traditional Religion (0.9%), or non-religious (3.0%). Regarding ethnic backgrounds, the Ga/Adangbe formed the largest group (46.2%), followed by the Akan (31.4%) and Ewe (10.8%), with other ethnicities (10.3%), Guan (0.9%), and Yoruba (0.5%) represented in smaller numbers.

### 3.2. TB Knowledge

Almost all participants recognised a persistent cough as a major symptom of TB. Approximately 62.2% of participants were aware that another disease, apart from COVID-19, causes a persistent cough, and 91.2% identified TB as that disease (Table 2). This was corroborated by qualitative accounts. One participant noted “*The characteristics and symptoms of TB. A TB patient has night sweats. The person coughs and grows slim. Within two weeks, the person will experience a drastic weight reduction. The person may even cough up blood if he or she doesn’t visit the hospital. Lung tissues will be seen in the blood*. [TB-DM IDI01_Female_Public_Health_Nurse: 50-50 (0)]”. Information sources varied, with most coming from radio, television, or newspapers (41.5%), followed by community discussions (39.7%) and information obtained from hospitals (27.2%).

While over half of the sample (51.5%) accurately identified bacteria as the cause of TB, a significant percentage (36.0%) were unsure, and 4.8% attributed it to supernatural causes. The bacterial cause of TB was supported by the qualitative accounts, as strikingly, a participant mentioned the name of the physician who first diagnosed an individual with the disease and the year the diagnosis was made, as evident in this illustrative quote:

*It is caused by Mycobacterium tuberculosis*. *That is the causative organism. It’s a bacterium that causes it. To my knowledge, it was diagnosed in 1882 by Dr. Robert Koch. This happened on the 24th of March. His investigation led to the discovery of the causative organism of TB.*

[TB-DM IDI01_Female_Public_Health_Nurse: 46-46].

Knowledge of TB transmission was relatively strong, as 82.4% reported airborne transmission through coughing or sneezing. This was corroborated by the qualitative accounts, as a participant indicated that “*When an infected person spits, coughs, or sneezes into the atmosphere, anyone around you with low immunity is at a very high risk of contracting the bacteria*. [IDI001_Female_NS: 26 26 (0)]”. Most participants (89.0%) viewed TB as life-threatening, while 87.9% believed that it is curable, primarily through hospital treatment (91.2%). However, misconceptions persisted, including beliefs related to spiritual or evil causes and various alternative treatment methods. Awareness of tuberculosis symptoms varied, with cough (79.4%) being the most recognised symptom, followed by weight loss, chest pain, and haemoptysis. Notably, 46.9% of the participants lacked adequate knowledge about TB, indicating a need for improved public health education programs.

### 3.3. Diabetes Knowledge

A significant percentage of the participants had either heard of or known something about DM (87.2%). Among those who were aware, 71.1% recognised a local name for the disease. Sources of information about diabetes varied, with knowing someone who has it (39.4%), community gossip (33.3%), and radio/TV/newspapers (37.3%) being common, while being a patient oneself was less common (8.7%); 65.9% of the participants identified nutritional factors as the primary cause of DM, followed by genetic predisposition at 34.4%. Corroboratively, it was unearthed by the in-depth interviews that eating heavy carbohydrate meals and retiring to bed immediately, as well as consuming too much sugary food without regular exercise, are potential causes of diabetes mellitus.

*When you continuously eat heavy food (highly dense carbohydrate food) and go to bed immediately, you may develop diabetes. So, it’s a cause of diabetes. It’s all about eating a lot of carbohydrates.*

[IDI001_Male_HS: 54-54 (0)].

*When someone takes in a lot of sugar and eats heavy foods late at night.*

[IDI002_Female_CM: 31-31 (0)].

Much lower percentages indicated interpersonal contact transmission (3.9%), water sources (1.6%), zoonotic transmission (0.5%), or environmental causes (1.3%), with a negligible minority citing insulin resistance (2.9%). A participant described diabetes succinctly as follows: “*Diabetes is a non-communicable disease. It is rather a lifestyle disease. It is based on how you live your life and how you eat. If an individual doesn’t eat well, he or she has a chance of developing diabetes. Exercising regularly will burn fat and increase blood circulation*. [IDI002_Male_NS-(0)]”. Furthermore, a nursing student considered diabetes as congenital, saying “*And as I said, with diabetes, some are congenital. Maybe some have a family history of diabetes, and some are from poor dietary intake* [IDI001_Female_NS: 26-26 (0)]”. This suggests that an individual whose parents are diabetic may also develop diabetes mellitus.

Most participants asserted that both sexes are equally affected (54.3%), while a significant portion perceived a higher prevalence among females (27.6%). Those aged 41 years and older were overwhelmingly considered to be the most affected age group (53.0%), considerably higher than the sum of all younger age groups (28.9%). The main signs and symptoms reported included excessive urination (28.9%), accidental weight loss (21.3%), the presence of sugar in urine (21.8%), fatigue (21.0%), increased thirst (18.1%), obesity (13.7%), and severe hunger (11.8%). A participant described the signs and symptoms of diabetes as follows: “*For diabetes, the person drinks so much water and suffers from weight loss. […]. Sometimes the person will have shivers. The person will also have dizziness and urinate a lot, especially at night*. [TB-DM IDI01_Female_Public_Health_Nurse: 53-53 (0)]”.

Over two-thirds of the participants (81.9%) perceived DM as life-threatening. Most believed that diabetes is manageable (68.5%), with the most common treatment cited as hospital care (66.7%), followed by local/home treatment (15.0%), pharmacy/drugstore (12.6%), and traditional/faith healers (4.5%). For the minority who thought that it was not curable, the explanation was that it is in the bloodstream (39.7%) (Table 3). Overall, the knowledge assessment revealed a slight divide between poor (48.7%) and good (51.3%) DM knowledge within the studied population.

## 4. Local Names for TB and Diabetes

Tuberculosis is known in Ewe as “Yɔmekpe”, which means “grave cough”. Considering the chronic nature of this cough and its associated excruciating pains, it is believed that it may lead patients to their graves. In Twi, tuberculosis is “Nsamanwa”, meaning “ghost cough”. TB is called Nsamanwa because it is associated with growing lean, which is construed as becoming a ghost. In Ga, TB is called “worlomofom”, which means “bad cough”.

*It is believed that in the past, if you had TB, you would cough till you died. The cough would lead to death. That is why they call it yɔmekpe, a grave cough. It is called Nsamanwa in Twi because you cough and become so lean. Like Saman, a ghost. That is how the name evolved*.

[TB-DM IDI01_Female_Public_Health_Nurse: 26-26 (0)].

*Tuberculosis is called “worlomofom” in the Ga language. Worlomo means “to cough,” and “fom” is “bad”*. *Therefore, “worlomofom” means “bad cough”.*

[IDI004_Female_CM: 10-10 (0)].

Diabetes mellitus is known in Twi as “asikyireyareɛ”, “sukluidɔ” or “sukluidɔlele” in Ewe, and *siklihila”* in Ga. In these three languages, diabetes mellitus means “sugar disease”, as articulated in the voices of these two participants:

*And then with diabetes, so we see that if someone has too much sugar, it’s a sugar disease. So that’s why we call it “*asikyireyareɛ*”.*

[IDI001_Female_NS: 18-18 (0)].

*Diabetes is called sukluidɔ in Ewe*.

[IDI001_Male_HS: 20-20 (0)].

*Diabetes is sukluidɔlele in Ewe. In Twi, they call it “*asikyireyareɛ*”.*

[TB-DM IDI01_Female_Public_Health_Nurse: 24-24 (0)].

*In Ga, Diabetes is called “siklihila”-sugar disease.*

[IDI004_Female_CM: 10-10 (0)].

## 5. TB and DM Prevention

Participants argued that conscious efforts to lose weight, exercising regularly, consuming less alcohol and starchy food, and eating more vegetables—including carrots, cucumbers, and cabbage—are plausible ways to avoid developing DM.

*Prevention, we have to be mindful of what enters our mouths. We eat too many sugary foods and starchy foods. That is the main basis. […]. Increase our physical activities. A lot of exercise is required to reduce your weight. This will prevent you from being at risk for diabetes. It doesn’t mean that slim people don’t develop diabetes. But from my observation, I’ve noticed that those who are slim also get diabetes. […]. We should reduce our intake of sugar and starchy foods and increase fruits and vegetables, especially green leafy vegetables, carrots, cucumbers, cabbage, kontomire [cocoyam leaf], aleifoo [African spinach], and bokoboko [waterleaf].*

[IDI01_Female_Public_Health_Nurse: 61-61 (0)].

*People can do daily or weekly check-ups of their sugar levels. I think eating vegetables, fruits, and limiting alcohol intake*.

[IDI004_Female_CM: 32-32 (0)].

Participants intimated that TB can be prevented through social distancing, proper handwashing, wearing nose masks, and avoiding crowds.

*Tuberculosis can be prevented through social distancing, proper handwashing, wearing nose masks, and avoiding overcrowded areas*.

[IDI002_Male_NS-35-35 (0)].

Furthermore, a participant averred that isolating TB patients may prevent others from contracting it.

*There should be isolation when a person is infected. The person should be isolated from unaffected individuals. Especially after the incubation period, so if you know you have it, you don’t want to be exposed to those who do not have it, to prevent the spread of TB. And those who have it should be treated*.

[IDI005_Male_HS: 29-29 (0)].

## 6. Stigmatisation Towards TB Patients

The study revealed complex perceptions of TB stigma. A slight majority (52.2%) indicated willingness to engage with individuals diagnosed with TB, while 47.8% said that they would not, citing fear of infection (93.8%) (Table 4); 79.1% of participants indicated that TB is contagious. Furthermore, 71.0% of participants opined that they were at risk of TB infection, reflecting widespread awareness of its transmissibility. TB stigma is largely driven by an exaggerated fear of contagion, as suggested by this participant:

*Since TB can be transmitted, people will distance themselves from you a little. […]. With DM, people will get closer to you because they know they will not be infected. So, that’s the difference*.

[IDI001_Male_HS: 107-107 (0)].

Emotional reactions to a hypothetical TB diagnosis varied: 39.4% expected sadness, 29.0% fear, and 19.7% surprise, while 7.3% indicated that they would feel ashamed or embarrassed. Most participants would inform family members (52.5%) or healthcare workers (47.9%) about such a diagnosis, whereas very few would disclose it to landlords, religious leaders, or keep it to themselves. Encouragingly, 79.9% would seek hospital care promptly upon experiencing symptoms, with only a minority (14.4%) waiting until other treatments had failed. Overall, the study found 44.6% of the participants to hold more stigmatising views, while 55.4% exhibited less stigmatisation, highlighting progress but also the need for focused anti-stigma interventions in TB education programs. The qualitative interviews revealed that patients internalise people’s reactions toward them, leading to their withdrawal from society, as articulated by these participants:

*They won’t be happy because they think they will use that to laugh at or insult them later. So, they prefer to keep it rather than discuss it with others*.

[IDI003_Female_HS: 93-93 (0)].

*Because it’s not something to be proud of, it’s not like most people feel that their illness is supposed to be confidential. Even sugar, diabetes that is not stigmatised, even they choose to keep it to themselves*.

[IDI004_Female_CM: 58-58 (0)].

## 7. Stigmatisation Towards DM Patients

Almost all participants (*n* = 435; 99.5%) considered diabetes less stigmatising, while a negligible percentage (0.5%) thought otherwise. In terms of interactions, most participants (83.1%) expressed their willingness to interact with DM patients, while a smaller percentage (16.9%) indicated otherwise. Of the 74 who chose not to interact, the main reason for their reluctance was fear of infection (78.4%), far exceeding the number who believed that patients were “looking sickly” (10.8%) or cited other reasons (12.2%). This fear of infection sharply contrasts with the common understanding of the non-communicability of DM; when asked if DM is a communicable disease, a clear majority (61.6%) responded “No” (Table 5).

### Coping with TB- and DM-Related Stigma

Participants opined that TB patients cope with stigma by self-isolating. It suffices that some patients adopt the strategy “out of sight, out of negative reactions from people” to manage their stigma experiences.

*Sometimes they even relocate to avoid negative reactions from people. But if it is at their own expense, they may have to pay rent at the new place, although they might not have paid rent at the previous places.*

[TB-DM IDI01_Female_Public_Health_Nurse: 134-134 (0)].

In addition, patients draw on entertaining activities, including playing online, watching funny videos, and reading books, to subvert their stigmatising experiences.

*Some people try to play online games, watch videos, read, or use social media. This may help them get their minds off this trouble. Reading, exercising, playing games, watching funny videos, and movies. Any of these things can help them*.

[IDI001_Male_HS: 135-135 (0)].

## 8. Stigma Implications

### 8.1. Suicidal Ideation

It was unearthed that stigma related to the diseases pushes patients to think of committing suicide. Unwelcoming remarks from community members, including name-calling and mocking, and the feeling of societal rejection, cause patients to nurse suicidal ideation in a bid to end their suffering, as communicated by this participant:

*If the patient is not firm, it will affect him or her. Some community members mock them. It feels like there is no one for them to talk to. You feel useless on this earth because of your condition. Yeah. So, some people, when you delve into most suicidal cases, you see that it’s because of the way we relate to them. It’s because they feel the absence of people around them. It appears to them that there is no one for them. It has always been their perception that life isn’t fair to them. They think it is better to die than to be alive*.

[IDI001_Male_HS: 131-131 (0)].

### 8.2. Social Participation

Diverse opinions were offered about the impact of stigma on patients’ presence at social gatherings. Some patients internalise the stigma, as they often anticipate that people may not accept them at social events, hence the decision to remain indoors.

*Patients’ social interactions may be affected. People aren’t able to attend programs due to their illnesses. They will be secluded indoors. They will not be able to interact with people. […]. A person who has TB will not be able to socialise like someone who has diabetes because people know that TB is airborne, so they wouldn’t want to interact with them.*

[TB-DM IDI01_Female_Public_Health_Nurse: 116-116 (0)].

However, another participant indicated that patients could attend social events without intimidation. This participant cited the former President of South Africa as a TB patient who mustered the courage to run for the presidency after recovering from the condition and won the elections massively. It suffices that a patient with a positive self-image and strong mental fortitude may not miss any social event due to the fear of being stigmatised, as mooted by this participant:

*I don’t think that one can affect you. TB is curable. If you are determined like the president of South Africa, Nelson Mandela, stigma cannot affect your political participation. He had TB, but later became the president of his country. I don’t think it will prevent patients from engaging in politics.*

[IDI001_Female_NS: 83-83 (0)].

### 8.3. Economic Implications

It was uncovered that while diabetes patients may not suffer economic loss due to stigma, TB patients experience it because of the fear of infecting people when they get closer to them. TB patients selling food items are not patronised, as expressed in the following voices:

*So, it affects them. It also lowers productivity. Because if I sell, and people get to know I have TB, and they also know TB is highly transmissible, they will not buy from me. My goods will rot.*

[IDI001_Female_NS: 81-81 (0)].

### 8.4. Religious Implications

Participants offered varied perspectives on the impact of stigma on patients’ religious participation. While some participants indicated that patients may face isolation at religious settings, restricting their involvement in such activities, others argued that stigma does not affect their participation in any way.

*In church, you may be isolated from people. This may prevent you from participating in church activities*.

[IDI005_Male_HS: 77-77 (0)].

### 8.5. Marital Implications

It was argued that since TB is highly communicable and DM is hereditary, unaffected individuals may fear marrying or remaining in marriages with patients, as articulated in the voices of these two participants.

*People think that if your parents have diabetes, you may also have it. It’s something generational. So, let’s say a man gets married to a woman and later finds out that she is diabetic, he will leave her because he doesn’t want his children to inherit the disease.*

[IDI001_Female_NS: 81-81 (0)].

*Yes, because since your partner knows you have TB and it is transmissible, he or she will break up with you. This is because when you sleep on the same bed with him or her and cough, he or she may be infected and probably die from it.*

[IDI005_Male_HS: 79-79 (0)].

### 8.6. Health Implications

Stigma associated with TB and DM may prevent patients from seeking biomedical treatment. Some patients perceived health personnel’s attitudes as those of the larger community and, therefore, would not visit health facilities for treatment. Such patients doubted whether personnel would keep their status a secret, as doing otherwise may attract stigmatising attitudes from the community.

*They may not want to discuss it. When community attitudes toward patients are positive, they encourage doctors to assist them. However, some patients worry that doctors will share their health status with others. This concern arises because doctors are similar to community members. It prompts them to stay home as their conditions worsen.*

[IDI001_Male_HS: 115-115 (0)].

It was further revealed that patients’ social interactions impact their treatment outcomes. It suffices that healthy interactions may facilitate speedy recovery, while stigmatising attitudes from unaffected individuals may prolong healing. This is because patients’ mental states also play critical roles in the healing process, as intoned by this participant:

*If you can connect with patients empathetically, despite their conditions, it can accelerate their recovery. A sound mind aids in a faster recovery for patients. […]. Stigma does not facilitate quick recovery. It rather adds to the problem. Overthinking can even increase stress levels or exacerbate the situation.*

[IDI001_Female_NS: 79-79 (0)].

## 9. Preventing TB- and DM-Related Stigma

Increased social support or capital for patients may reduce TB- and diabetes-mellitus-related stigma. Making patients feel loved by people within their social networks offers them a sense of belonging, and receiving empathy during their illness may enhance their social participation. Providing patients with psychosocial support and also educating community members about the transmission modes of TB and its curability may reduce its related stigma.

*The person should be treated kindly. They should be cared for and not made to feel inferior or useless. They shouldn’t be demeaned or made to feel disgusted about their disease. Instead, they should bring people closer and support them in any way they can. They shouldn’t gossip about the person; rather, they should assist him or her to receive treatment.*

[IDI001_Male_HS: 97-97 (0)].

*Educating families and communities that tuberculosis is curable and isn’t a chronic disease, but an infectious disease, will help patients access proper care to return to their normal lives*.

[IDI002_Male_NS-81-81 (0)].

## 10. Treatment-Seeking Behaviour

More than half of the participants (66.6%) perceived treatment cost as a major barrier to seeking biomedical care. Other significant barriers included transportation cost (21.5%) and “other reasons” (20.1%), while distance from health facilities (14.0%), poor quality of care (10.3%), and rude behaviour of the health staff (10.1%) were cited less frequently. For instance, a participant opined that “*Most people don’t like the hospital, and it’s because of some behaviours of the health staff. Some of them are too arrogant. Consequently, they’ll prefer the native doctors. At least, the healer will give them some herbs to take. This may not come at any cost. However, when I visit the hospital, I will be asked to pay for treatment and medications. This may cost a lot. So, they would prefer to see native doctors rather than seek hospital care*. [IDI001_Female_NS: 61-61 (0)]”.

Further exploring why patients choose traditional, herbal, or faith healers instead of healthcare facilities, treatment costs (44.9%) emerged as a significant factor. For instance, a participant indicated that treatment costs at traditional therapeutic settings are relatively cheaper than health facilities: “*It is money that you will take to the hospital, so if you don’t have it, you will prefer to see a herbalist to seek care. The herbalist’s medicine will not cost millions. If there is money, you go straight to the hospital* [IDI002_Female_CM: 69-69 (0)]”. Quality of care was also a significant reason (22.4%), with a large number of respondents listing “others” (24.5%) as reasons for using alternative healers. Transportation cost (16.0%), proximity (9.8%), and a perceived lower level of stigma (11.9%) were also mentioned as common reasons for utilising traditional/other healers (Table 6).

Furthermore, participants indicated that TB and DM patients receive treatment from diverse care cascades. While some participants asserted that diabetes patients seek biomedical care, others maintained that individuals with TB seek alternative care.

*From my experience, those with diabetes mostly go to the health facility. But those with TB mostly seek care from these native doctors. That’s what I’ve seen before*.

[IDI001_Female_NS: 58-58 (0)].

*Most seek treatment from health facilities.*

[IDI002_Male_NS: 45-45 (0)].

Participants provided various reasons that facilitate patients’ choice of one care option over the other. Patients seek biomedical care because they trust the effectiveness of care provided at health facilities.

*They mostly seek care from health facilities because they trust their diagnosis and treatment*.

[IDI002_Male_NS: 47-47 (0)].

However, it was unearthed that some patients would prefer traditional healing to biomedical care because of the stigma associated with their conditions. Some patients articulated the view that their families and other close relations may be informed about their health status if care is sought from biomedical settings, as intimated by this participant:

*A person may choose home-based care over biomedical care because of the stigma associated with the disease, or he or she doesn’t want his or her family to know about the condition, so he or she will visit a traditional healer for treatment. The person will not go to the health facility for proper diagnosis and treatment*.

[IDI002_Male_NS: 49-49 (0)].

Furthermore, it was revealed that patients’ choice of traditional healing over biomedical care is influenced by successful treatment testimonies shared by people within their social networks. It was also revealed that patients are discouraged from seeking biomedical care if their peers share unsuccessful stories with them, as articulated by this participant:

*Some individuals influence others to seek treatment from traditional healers. For example, a friend might go to the hospital for treatment, but if it doesn’t work and then tries herbal medicine, and it works, this may lead the friend to seek care from a traditional healer. For instance, I was also dealing with diabetes and other issues, but when I tried herbal medicine, it worked for me, so you might consider trying it too. This encourages people to choose traditional ways of managing their condition over biomedical care.*

[IDI005_Male_HS: 59-61 (0)].

### 10.1. Multivariate Analysis Between Participants’ Sociodemographic Profiles and Knowledge of TB

The analysis explored the relationship between participants’ sociodemographic profiles and their knowledge about TB. Participants aged 60 and older were strongly associated with good knowledge, with an adjusted odds ratio of 3.377 (95% CI: 1.525–7.475, *p* = 0.026). Educational level was also significantly related to knowledge. Participants with tertiary education were 3.074 times more likely to have good knowledge than those with only basic education (95% CI: 1.664–5.678, *p* < 0.001) (Table 7). Conversely, certain occupations were strongly linked to poor knowledge: unemployed individuals were less likely than civil servants to have good knowledge, with an adjusted odds ratio of 0.079 (95% CI: 0.009–0.697, *p* = 0.022). Other sociodemographic factors, such as sex, education level (no education or secondary education), types of occupation (self-employed, students), religion (Christian, Islam, and none), and ethnicity (Ewe, Ga/Adangbe, Guan, and other ethnicities), did not show a significant relationship with knowledge level in the adjusted analysis at the *p* < 0.05 level.

### 10.2. Relationship Between Participants’ Sociodemographic Profiles and TB Stigma

Logistic regression analysis of the association between participants’ profiles and stigma towards TB patients revealed that males were 1.634 times more likely to stigmatise TB patients compared to their female counterparts (95% CI: 1.013–2.634, *p* = 0.044) (Table 8). Participants aged 36–59 (aOR 0.354, 95% CI: 0.202–0.619, *p* < 0.001) were more likely to stigmatise TB patients compared to the 18–35 years age group. Educational level was also significantly related to TB stigma. Participants with tertiary education were 5.522 times more likely to stigmatise than those with only basic education (95% CI: 2.817–10.823, *p* < 0.001). Occupation, however, had robust significant associations relative to civil servants, with the self-employed (aOR 65.689, 95% CI: 7.187–600.380, *p* < 0.001), students (aOR 13.942, 95% CI: 1.551–125.303, *p* = 0.019), and unemployed persons (aOR 59.885, 95% CI: 6.315–567.893, *p* < 0.001) all being significantly more likely to show patients stigmatising attitudes. Religious affiliations such as Christianity and Islam showed no strong relations with stigmatising attitudes compared to the African Traditional religion category in the adjusted analysis. Finally, among ethnic groups, the Ga/Adangbe group had considerably higher odds of presence of stigma compared to the Akan group (aOR 2.26, 95% CI: 1.359–3.773, *p* = 0.002), but other ethnic groups, such as Ewe, Guan, and other ethnicities, did not show strong associations.

### 10.3. Relationship Between Participants’ Sociodemographic Profiles and Knowledge of DM

Logistic regression analysis of the association between participants’ sociodemographic profiles and knowledge about DM indicated that males had 0.601 times the odds of being knowledgeable about DM compared with females (95% CI: 0.387–0.933, *p* = 0.023) (Table 9). Age groups did not show a statistically significant association with knowledge about DM. Individuals with tertiary education were 4.063 times more likely to have good knowledge of DM (95% CI: 2.177–7.580, *p* <0.001).

Furthermore, none of the other occupational categories (retired/pensioner, self-employed, student, and unemployed) compared with civil servants showed a statistically significant association with the odds of good diabetes knowledge in the adjusted analysis. Although the self-employed (cOR = 0.186, *p* = 0.032) and unemployed (cOR = 0.166, *p* = 0.027) groups had significantly lower odds of good knowledge in the crude analysis, these associations became non-significant after adjustment (aOR = 0.385, *p* = 0.274 for self-employed; aOR = 0.251, *p* = 0.124 for unemployed). Regarding ethnicity, Ewes displayed significantly lower odds of good DM knowledge, with an adjusted odds ratio of 0.341 (95% CI: 0.164–0.711, *p* = 0.004). Similarly, the “other ethnicities” group had significantly lower odds of good DM knowledge (aOR = 0.384, *p* = 0.043).

## 11. Discussion

Our study investigated community knowledge, stigma, and health-seeking behaviour related to TB and DM in Ghana. Participants’ sociodemographic factors (sex, age, education, and occupation) were associated with their understanding of TB and DM. Older participants aged 60 years and above demonstrated a better understanding of the diseases than the younger ones. Individuals with tertiary education were 2.974 times and 2.680 times more likely to have good knowledge about TB and DM, compared to individuals with only basic education (95% CI: 1.472–6.008, *p* = 0.002 and 95% CI: 1.333–5.385, *p* = 0.006, respectively). This supports other research findings indicating that higher education levels and older age are associated with better knowledge of the causes, signs, symptoms, transmission, and prevention of TB and DM [23,24,25,26,27]. For example, most participants viewed TB (89.0%) and DM (81.9%) as life-threatening and believed that they are treatable with biomedical methods. As noted in other studies, our findings show that TB is transmitted when an infected person spits, coughs, yawns, or sneezes into the air, and others nearby inhale it [28,29]. This study, however, revealed that quite a significant number of individuals (46.9%) lacked knowledge about TB. This might have been because knowledge assessment extended beyond recognition of common symptoms to include understanding of transmission, prevention, treatment duration, and curability. It might equally stem from the fact that many participants were familiar with the widely publicised symptoms of TB, such as a persistent cough, but lacked adequate knowledge in other domains of the condition.

Our study found that participants perceived DM as a disease caused by an unhealthy lifestyle, leading to the accumulation of excess sugar in the bloodstream, which is why it is called sukluidɔ/suklui dɔlele (sugar disease), asikyireyareɛ (sugar disease), and siklihila (sugar disease) in the Ewe, Twi, and Ga languages, respectively. It was also indicated that consuming sugary, carbohydrate-rich foods and alcohol could lead to DM. This finding aligns with those of other studies in Ghana [30,31,32,33]. Its symptoms were said to include frequent urination, weight loss, the presence of sugar in urine, fatigue, increased thirst, obesity, dizziness, and severe hunger. A large percentage of respondents viewed diabetes as life-threatening (81.9%). This suggests that individuals may not develop DM if they engage in a healthy lifestyle, such as consuming less sugary food and exercising regularly. It also confirms the need for intensified efforts in community education.

The major TB symptom described was excessive coughing, which could be fatal if not treated early. This is why it is called “*nsamanwa*” (ghost cough), “*yɔmekpe*” (grave cough), and “*worlomofom*” (bad cough) in the Twi, Ewe, and Ga languages, respectively. This suggests that community members understood that TB is a life-threatening disease that requires prompt medical attention to prevent patients from experiencing health complications. This perception may fuel social isolation and discrimination against patients. Our study reveals that the participants understood that TB is curable and airborne, but exhibited stigmatising attitudes toward patients. It was further revealed that males were 1.668 times less likely to stigmatise TB patients compared to their female counterparts (95% CI: 1.013–2.746, *p* = 0.044). Participants aged 36–59 (aOR 0.354, 95% CI: 0.202–0.619, *p* < 0.001) were more likely to stigmatise TB patients compared to the 18–35 age group. This suggests that knowledge about the curability or mode of transmission alone may be insufficient to change these attitudes. This finding reaffirms other studies showing that stigma against TB patients festers in family, societal, and therapeutic settings [34,35,36,37,38,39].

TB stigma is engineered by fear of infection, which disrupts patients’ meaningful social interactions. This supports the findings of other studies showing that TB patients avoid social interactions due to the fear of infecting others [34,40,41,42,43]. As observed in other studies, we found stigma to impact patients negatively, including constricted social participation, economic loss, suicidal ideation, and reluctance to seek biomedical treatment [12,41,42,44,45,46,47,48,49,50,51]. TB-related stigma may be alleviated through community education and enhanced social support for patients. This supports other studies that proposed health education as a crucial intervention for increasing community knowledge about TB and its implications for patients and their carers [52,53,54]. Once community members have become health-literate, they may display positive attitudes toward patients, which could improve their social participation. However, this study found stigma to be almost absent in DM, which contradicts many other studies showing that DM stigma adversely affects relationships and support in healthcare settings [55], along with access to quality healthcare [30,34,55]. It appears that participants perceived DM as less stigmatising compared to TB because it cannot be transmitted from one person to another.

Our study found that treatment costs, transportation costs, long distances, stigma (such as hostile attitudes from healthcare professionals), and the perceived effectiveness of traditional healing influence patients’ choice of traditional remedies and other alternative care options over biomedical care. This partially supports the argument that the healthcare system involves multiple players and that people’s cultural beliefs and practices affect their health-seeking behaviour [56]. It also confirms the findings of other studies, which indicated that financial constraints, lack of knowledge, mistaken beliefs, and misconceptions about causes, modes of transmission, symptoms, and treatment, along with stigma, not only influence TB and DM patients’ healthcare decisions [11,12,13,14,15,16,47,57,58,59,60] but also increase their preference for alternative care, such as spiritual, faith-based, and traditional healing, over biomedical care [17,18]. This leads to poor treatment adherence, delays in diagnosis, and a higher spread of TB within the community [19]. These findings highlight barriers that policymakers and implementers must address to ensure that patients receive quality healthcare.

We acknowledge that the use of convenience sampling and a relatively small number of actual TB patients for the in-depth interviews might have limited the depth and reliability of the qualitative data.

## 12. Conclusions

Community members demonstrated appreciable knowledge about TB and DM, including causes, signs and symptoms, modes of transmission and prevention, and their life-threatening nature. Our study reveals that social determinants of health, including poverty, local beliefs, stigma, and poor attitudes among healthcare professionals, compel patients to seek traditional remedies rather than biomedical care. While our study reveals the presence of significant stigma in TB, the exact opposite appears in the case of diabetes mellitus. Awareness of the diseases and the availability of effective treatment at health facilities need to be intensified across the country. Conscious efforts to break down cultural barriers and reduce the financial costs of care may drive more TB and DM patients toward biomedical care. In addition, interventions to improve physician–patient relationships may increase patients’ interest in biomedical care, as the unwelcoming behaviour of healthcare providers emerged as a significant impediment to seeking care from health facilities. We recommend that the national TB and DM control programs coordinate advocacy, communication, and social mobilisation activities across the country to improve community knowledge (including patients), attitudes, and practices, and to reduce misconceptions about TB transmission and DM development.

## Figures and Tables

**Table 1:** Participants’ sociodemographic characteristics.

Characteristics	Frequency (*n*)	Percentage (%)
**Sex**		
Female	284	65.0
Male	153	35.0
**Age**		
18–35	243	55.6
36–59	144	33
60+	50	11.4
**Educational Status**		
No education	49	11.2
Basic education	184	42.1
Secondary education	107	24.5
Tertiary/graduate	97	22.2
**Occupation**		
Civil servant	12	2.8
Retired/pensioner	7	1.6
Self-employed	255	58.4
Student	99	22.6
Unemployed	64	14.6
**Religion**		
African Traditional Religion	4	0.9
Christianity	372	85.1
Islam	48	11.0
None	13	3.0
**Ethnicity**		
Akan (Asante/Fante/Akuapim/Bono)	137	31.4
Ewe	47	10.7
Ga/Adangbe	202	46.2
Guan	4	0.9
Yoruba	2	0.5
Other ethnicities	45	10.3

**Table 2:** Participants’ knowledge about TB.

Characteristics	Frequency (*n* = 437)	Percentage (%)
**Have you heard or know of any disease that makes you cough for a very long time (Not COVID-19)**		
No	165	37.8
Yes	272	62.2
**What is the name of that disease**	***n*** ** = 272**	
No idea	8	2.9
Other	16	5.9
Tuberculosis	248	91.2
**How come you know of these diseases**	***n*** ** = 272**	
I was/am a patient	4/272	1.5
I know someone who had it	70/272	25.7
Community members talk about it	108/272	39.7
Radio/TV/Newspaper	113/272	41.5
From hospital staff/Posters	74/272	27.2
Others	25/272	9.2
**What is/are the cause(s) of tuberculosis**		
Bacteria/germ	140/272	51.5
Witchcraft/spirit/curse	13/272	4.8
Don’t know	98/272	36.0
Other causes	29/272	10.7
**How is TB transmitted**		
Through the air when an infected person coughs/sneezes	224/272	82.4
Living with a TB patient	56/272	20.6
Sharing of dishes/plates	41/272	15.1
Smoking and drinking alcohol	26/272	9.6
Don’t know	21/272	7.7
Others	7/272	2.6
**How serious is TB**	***n*** ** = 272**	
Don’t know	17	6.3
Life-threatening	242	89.0
Not very serious	13	4.8
**Which gender do you think is most affected by TB**		
Both	137	50.4
Women	21	7.7
Men	72	26.5
No idea	42	15.4
**Which age group do you think is most affected by TB**		
All age groups	113	41.5
Children (0–18 years)	18	6.6
No idea	14	5.2
Older adults (41 and above)	79	29.0
Young adults (26–40 years)	31	11.4
Youth (19–25 years)	17	6.3
**What are some of the signs and symptoms of TB**		
Cough	216/272	79.4
Night sweat	55/272	20.2
Bloody sputum	71/272	26.1
Weight loss	88/272	32.4
Chest pain	85/272	31.3
Fatigue	59/272	21.7
Shortness of breath	36/272	13.2
Don’t know	26/272	9.6
Other symptoms	12/272	4.4
**Do you think TB is curable/treatable**		
I don’t know	22	8.1
No	11	4.0
Yes	239	87.9
**How can TB be cured**	***n*** ** = 239**	
Herbs	19/239	8.0
Hospital (Biomedical)	218/239	91.2
Faith healer	12/239	5.0
Pharmacy (drugstore)	6/239	2.5
Don’t know	9/239	3.8
Others	2/239	0.8
**Why is TB not curable**	***n*** ** = 11**	
Spiritual disease	3/11	27.3
Cursed disease	1/11	9.1
In the blood	3/11	27.3
Others	6/11	54.6
**Cost of TB treatment in the country**	***n*** ** = 272**	
Don’t know	81	29.8
It is free	117	43.0
Pay a fee	74	27.2

**Table 3:** Participants’ knowledge about DM.

Characteristics	Frequency (*n* = 437)	Percentage (%)
**Have you heard of or know of a disease called Diabetes**		
No	56	12.8
Yes	381	87.2
**What is its local name**	***n*** ** = 381**	
**Asikyire yareε/siklihela (sugar disease)**		
No	110	28.9
Yes	271	71.1
**No idea**		
No	277	72.7
Yes	104	27.3
**Other names**		
No	372	97.6
Yes	9	2.4
**How did you come to know of Diabetes**		
**I’m a patient**		
No	348	91.3
Yes	33	8.7
**know someone who had it**		
No	231	60.6
Yes	150	39.4
**Community members talk about it**		
No	254	66.7
Yes	127	33.3
**Radio/TV/newspapers**		
No	239	62.7
Yes	142	37.3
**Hospital staff/posters**		
No	312	81.9
Yes	69	18.1
**Other means**		
No	367	96.3
Yes	14	3.7
**How does one get diabetes**		
**Person to person**		
No	366	96.1
Yes	15	3.9
**Hereditary**		
No	250	65.6
Yes	131	34.4
**Dietary**		
No	130	34.1
Yes	251	65.9
**Waterbodies**		
No	375	98.4
Yes	6	1.6
**Animal person**		
No	379	99.5
Yes	2	0.5
**In the environment**		
No	376	98.7
Yes	5	1.3
**Insulin resistance**		
No	370	97.1
Yes	11	2.9
**No idea**		
No	346	90.8
Yes	35	9.2
**Other**		
No	344	90.3
Yes	37	9.7
**Which gender do you think is most affected by diabetes**		
Both	207	54.3
Women	105	27.6
Men	36	9.4
No idea	33	8.7
**Which age group do you think is most affected by diabetes**		
All age groups	110	28.9
Children (0–18 years)	20	5.3
Older adults (41 years and above)	202	53
Young adults (26–40 years)	34	8.9
Youth (19–25 years)	15	3.9
**What are some of the signs and symptoms of diabetes**		
**Obesity**		
No	329	86.4
Yes	52	13.6
**Increased thirst**		
No	312	81.9
Yes	69	18.1
**Frequent urination**		
No	271	71.1
Yes	110	28.9
**Extreme hunger**		
No	336	88.2
Yes	45	11.8
**Unintentional weight loss**		
No	300	78.7
Yes	81	21.3
**Fatigue**		
No	301	79
Yes	80	21
**Sugary urine**		
No	298	78.2
Yes	83	21.8
**No idea**		
No	276	72.4
Yes	105	27.6
**How serious is Diabetes**		
Don’t know	33	8.7
Life threatening	312	81.9
Not very serious	36	9.4
**Do you think Diabetes is manageable**		
Don’t know	52	13.6
No	68	17.8
Yes	261	68.5
**How can Diabetes be managed**		
**Local treatment/Home treatment (herbs)**		
No	324	85
Yes	57	15
**Traditional/faith healers**		
No	364	95.5
Yes	17	4.5
**Pharmacy/Drugstore**		
No	333	87.4
Yes	48	12.6
**Drugs specifically for TB Hospital (Biomedical)**		
No	127	33.3
Yes	254	66.7
**I don’t know**		
No	315	82.7
Yes	66	17.3
**If NO, why is Diabetes not manageable**	***n*** ** = 68**	
**Spiritual diseases**		
No	64	94.1
Yes	4	5.9
**Cursed diseases**		
No	66	97.1
Yes	2	2.9
**It’s in the blood of the person**		
No	41	60.3
Yes	27	39.7
**No idea**		
No	41	60.3
Yes	27	39.7

**Table 4:** Tabulation of TB stigma interaction.

Characteristics	Frequency (*n*)	Percentage (%)
**Would you interact with a patient with Tuberculosis**	***n*** ** = 435**	
No	208	47.8
Yes	227	52.2
**If no, what is your reason for not wanting to interact with them**	***n*** ** = 208**	
**Fear infection**		
No	13	6.3
Yes	195	93.7
**Person looking sickly**		
No	200	96.1
Yes	8	3.9
**Other reasons**		
No	197	94.7
Yes	11	5.3
**Would you regard TB as a communicable disease**	***n*** ** = 436**	
I have no idea	51	11.7
No	40	9.2
Yes	345	79.1
**Do you think you can get TB**	***n*** ** = 435**	
No	126	29.0
Yes	309	71.0
**How would you feel if you found you had TB**	***n*** ** = 259**	
**Fear/scared**		
No	184	71.0
Yes	75	29.0
**Surprised**		
No	208	80.3
Yes	51	19.7
**Ashamed/embarrassed**		
No	240	92.7
Yes	19	7.3
**Sad**		
No	157	60.6
Yes	102	39.4
**As normal, when I am sick**		
No	197	76.1
Yes	62	23.9
**Don’t know**		
No	245	94.6
Yes	14	5.4
**Who would you talk to when you have TB**	***n*** ** = 259**	
**Health worker**		
No	135	52.1
Yes	124	47.9
**family**		
No	123	47.5
Yes	136	52.5
**landlord**		
No	246	95.0
Yes	13	5.0
**Religious leader**		
No	253	97.7
Yes	6	2.3
**No one**		
No	247	95.4
Yes	12	4.6
**Others**		
No	256	98.8
Yes	3	1.2
**At what point will you go to the hospital**		
Other. Please specify…	14	3.2
When home/self-treatment/traditional treatment fails	63	14.4
Will go immediately	349	79.9
Won’t go at all	11	2.5

**Table 5:** Tabulation of DM stigma interaction.

Characteristics	Frequency (*n* = 437)	Percentage (%)
**Would you interact with patients with DM**		
No	74	16.9
Yes	363	83.1
**If no, what is your reason for not wanting to interact with them**	***n*** ** = 74**	
**Fear of being infected**		
No	16	21.6
Yes	58	78.4
**Patients looking sickly**		
No	66	89.2
Yes	8	10.8
**Others**		
No	65	87.8
Yes	9	12.2
**Would you regard Diabetes as a communicable disease**	***n*** ** = 437**	
No	269	61.6
No idea	98	22.4
Yes	70	16.0
Overall stigma towards diabetes		
More stigmatising	2	0.5
Less stigmatising	435	99.5

**Table 6:** Participants’ opinions about TB and DM health-seeking behaviour.

Characteristics	Frequency (*n* = 437)	Percentage (%)
**What are the impediments to TB and Diabetes patients seeking healthcare from the health facilities**		
**Treatment cost**		
No	146	33.4
Yes	291	66.6
**Distance to health facility**		
No	376	86.0
Yes	61	14.0
**Transportation cost**		
No	343	78.5
Yes	94	21.5
**Bad attitude of health personnel**		
No	393	89.9
Yes	44	10.1
**Poor quality of care**		
No	392	89.7
Yes	45	10.3
**Other reasons**		
No	349	79.9
Yes	88	20.1
**What makes TB or Diabetes patients choose to receive treatment from the traditional healer/herbal/faith healers over health facilities**		
**Treatment costs**		
No	241	55.2
Yes	196	44.8
**Transportation cost**		
No	367	84
Yes	70	16
**Proximity**		
No	394	90.2
Yes	43	9.8
**Quality of care**		
No	339	77.6
Yes	98	22.4
**Less stigmatising**		
No	385	88.1
Yes	52	11.9
**Others**		
No	330	75.5
Yes	107	24.5

**Table 7:** Association between participants’ sociodemographic profiles and knowledge of TB.

Characteristics	Poor Knowledge *n* (%)	Good Knowledge *n* (%)	cOR (95% CI)	*p*-Value	aOR (95% CI)	*p*-Value
**Sex**						
Female	125 (44.0%)	159 (56.0%)	1		1	
Male	80 (52.3%)	73 (47.7%)	0.717 (0.484–1.064)	0.099	0.728 (0.468–1.135)	0.161
**Age groups**						
18–35	125 (51.4%)	118 (48.6%)	1		1	
36–59	65 (45.1%)	79 (54.9%)	1.287 (0.851–1.947)	0.231	1.962 (1.164–3.309)	**0.011**
60+	15 (30.0%)	35 (70.0%)	2.472 (1.284–4.759)	0.007	3.377 (1.525–7.475)	**0.003**
**Educational level**						
Basic education	100 (54.4%)	84 (45.6%)	1		1	
No education	29 (59.2%)	20 (40.8%)	0.821 (0.433–1.556)	0.545	0.909 (0.445–1.860)	0.795
Secondary education	48 (44.9%)	59 (55.1%)	1.463 (0.907–2.362)	0.119	1.684 (0.989–2.869)	0.055
Tertiary/graduate	28 (28.9%)	69 (71.1%)	2.934 (1.733–4.966)	**<0.001**	3.074 (1.664–5.678)	**<0.001**
**Occupation**						
Civil servant	1 (8.3%)	11 (91.7%)	1		1	
Retired/pensioner	0 (0.0%)	7 (100.0%)	1		1	
Self-employed	123 (48.2%)	132 (51.8%)	0.098 (0.012–0.767)	**0.027**	0.177 (0.021–1.512)	0.114
Student	40 (40.4%)	59 (59.6%)	0.134 (0.017–1.08)	0.059	0.320 (0.036–2.811)	0.304
Unemployed	41 (64.1%)	23 (35.9%)	0.051 (0.006–0.421)	**0.006**	0.079 (0.009–0.697)	**0.022**
**Religion**						
African Traditional	3 (75.0%)	1 (25.0%)	1		1	
Christianity	164 (44.1%)	208 (55.9%)	3.805 (0.392–36.918)	0.249	3.491 (0.321–37.934)	0.304
Islam	27 (56.2%)	21 (43.8%)	2.333 (0.226–24.076)	0.477	1.907 (0.163–22.249)	0.606
None	11 (84.6%)	2 (15.4%)	0.545 (0.036–8.27)	0.662	0.556 (0.031–9.827)	0.689
**Ethnicity**						
Akan (Asante/Fante/Akuapim)	56 (40.9%)	81 (59.1%)	1		1	
Ewe	24 (51.1%)	23 (48.9%)	0.663 (0.341–1.289)	0.225	0.572 (0.275–1.189)	0.134
Ga/Adangbe	104 (51.5%)	98 (48.5%)	0.651 (0.42–1.01)	0.055	0.830 (0.514–1.339)	0.445
Guan	1 (25.0%)	3 (75.0%)	2.074 (0.21–20.454)	0.532	0.567 (0.041–7.854)	0.672
Other ethnicities	20 (44.4%)	25 (55.6%)	0.864 (0.438–1.705)	0.674	1.617 (0.613–4.264)	0.331
Yoruba	0 (0.0%)	2 (100.0%)	1		1	

Bold values indicate statistical significance at the *p* < 0.05 level.

**Table 8:** Association between participants’ sociodemographic profiles and TB stigma.

Characteristics	Absence of Stigma *n* (%)	Presence of Stigma *n* (%)	cOR (95% CI)	*p*-Value	aOR (95% CI)	*p*-Value
**Sex**						
Female	131 (46.1%)	153 (53.9%)	1		1	
Male	64 (41.8%)	89 (58.2%)	1.191 (0.801–1.771)	0.389	1.634 (1.013–2.634)	**0.044**
**Age groups**						
18–35	91 (37.45%)	152 (62.55%)	1		1	
36–59	71 (49.3%)	73 (50.7%)	0.616 (0.406–0.934)	**0.023**	0.354 (0.202–0.619)	**<0.001**
60+	33 (66.0%)	17 (34.0%)	0.308 (0.163–0.585)	**<0.001**	0.171 (0.076–0.384)	**<0.001**
**Educational level**						
Basic education	102 (55.4%)	82 (44.6%)	1		1	
No education	21 (42.9%)	28 (57.1%)	1.659 (0.878–3.133)	0.119	1.889 (0.912–3.912)	0.087
Secondary education	39 (36.4%)	68 (63.6%)	2.169 (1.33–3.538)	**0.002**	1.652 (0.947–2.881)	0.077
Tertiary/graduate	33 (34.0%)	64 (66.0%)	2.412 (1.447–4.021)	**0.001**	5.522 (2.817–10.823)	**<0.001**
**Occupation**						
Civil servant	11 (91.7%)	1 (8.3%)	1		1	
Retired/pensioner	5 (71.4%)	2 (28.6%)	4.4 (0.319–60.614)	0.268	20.877 (1.239–351.665)	**0.035**
Self-employed	104 (40.8%)	151 (59.2%)	15.971 (2.031–125.597)	**0.008**	65.689 (7.187–600.380)	**<0.001**
Student	55 (55.6%)	44 (44.4%)	8.8 (1.094–70.803)	**0.041**	13.942 (1.551–125.303)	**0.019**
Unemployed	20 (31.2%)	44 (68.8%)	24.2 (2.922–200.458)	**0.003**	59.885 (6.315–567.893)	**<0.001**
**Religion**						
African Traditional Religion	1 (25.0%)	3 (75.0%)	1		1	
Christianity	160 (43.0%)	212 (57.0%)	0.442 (0.046–4.286)	0.481	0.328 (0.025–4.380)	0.399
Islam	28 (58.3%)	20 (41.7%)	0.238 (0.023–2.459)	0.228	0.252 (0.017–3.744)	0.317
None	6 (46.2%)	7 (53.8%)	0.389 (0.032–4.796)	0.461	0.220 (0.012–3.927)	0.303
**Ethnicity**						
Akan (Ashanti/Fante/Akuapim)	67 (48.9%)	70 (51.1%)	1		1	
Ewe	23 (48.9%)	24 (51.1%)	0.999 (0.515–1.938)	0.997	1.419 (0.666–3.021)	0.365
Ga/Adangbe	71 (35.1%)	131 (64.9%)	1.766 (1.135–2.748)	**0.012**	2.264 (1.359–3.773)	**0.002**
Guan	3 (75.0%)	1 (25.0%)	0.319 (0.032–3.144)	0.328	0.573 (0.041–8.073)	0.68
Other ethnicities	29 (64.4%)	16 (35.6%)	0.528 (0.263–1.059)	0.072	0.506 (0.193–1.331)	0.168
Yoruba	2 (100.0%)	0 (0.0%)	1	.	1	

Bold values indicate statistical significance at the *p* < 0.05 level.

**Table 9:** Association between participants’ sociodemographic variables and knowledge of DM.

Characteristics	Poor Diabetes Knowledge *n* (%)	Good Diabetes Knowledge *n* (%)	cOR (95% Cl)	*p*-Value	aOR (95% CI)	*p*-Value
**Sex**						
Female	124 (43.7%)	160 (56.3%)	1		1	
Male	89 (58.2%)	64 (41.8%)	0.557 (0.374–0.829)	**0.004**	0.601 (0.387–0.933)	**0.023**
**Age groups**						
18–35	122 (50.21%)	121 (49.79%)	1		1	
36–59	69 (47.9%)	75 (52.1%)	1.096 (0.726–1.655)	0.663	1.632 (0.974–2.735)	0.063
60+	22 (44.0%)	28 (56.0%)	1.283 (0.696–2.367)	0.425	1.598 (0.746–3.424)	0.228
**Educational level**						
Basic education	100 (54.4%)	84 (45.6%)	1	.	1	
No education	29 (59.2%)	20 (40.8%)	0.821 (0.433–1.556)	0.545	0.840 (0.417–1.693)	0.627
Secondary education	62 (57.9%)	45 (42.1%)	0.864 (0.534–1.398)	0.552	0.936 (0.556–1.575)	0.802
Tertiary/graduate	22 (22.7%)	75 (77.3%)	4.058 (2.326–7.082)	0	4.063 (2.177–7.580)	**<0.001**
**Occupation**						
Civil servant	2 (16.7%)	10 (83.3%)	1		1	
Retired/pensioner	2 (28.6%)	5 (71.4%)	0.5 (0.054–4.672)	0.543	0.751 (0.058–9.792)	0.827
Self-employed	132 (51.8%)	123 (48.2%)	0.186 (0.04–0.868)	**0.032**	0.385 (0.070–2.128)	0.274
Student	42 (42.4%)	57 (57.6%)	0.271 (0.056–1.304)	0.103	0.521 (0.089–3.042)	0.469
Unemployed	35 (54.7%)	29 (45.3%)	0.166 (0.034–0.817)	**0.027**	0.251 (0.043–1.463)	0.124
**Religion**						
African Traditional	2 (50.0%)	2 (50.0%)	1		1	
Christian	177 (47.6%)	195 (52.4%)	1.102 (0.154–7.904)	0.923	0.791 (0.102–6.118)	0.822
Islam	26 (54.2%)	22 (45.8%)	0.846 (0.11–6.511)	0.873	1.189 (0.141–10.032)	0.873
None	8 (61.5%)	5 (38.5%)	0.625 (0.065–5.966)	0.683	0.672 (0.063–7.196)	0.742
**Ethnicity**						
Akan (Twi/Fante/Akuapim)	51 (37.2%)	86 (62.8%)	1		1	
Ewe	28 (59.6%)	19 (40.4%)	0.402 (0.204–0.793)	**0.008**	0.341 (0.164–0.711)	**0.004**
Ga/Adangbe	109 (54.0%)	93 (46.0%)	0.506 (0.325–0.788)	**0.003**	0.647 (0.402–1.042)	0.073
Guan	0 (0.0%)	4 (100.0%)	1		1	
Other ethnicities	25 (55.6%)	20 (44.4%)	0.474 (0.24–0.939)	**0.032**	0.384 (0.152–0.971)	**0.043**
Yoruba	0 (0.0%)	2 (100.0%)	1		1	

Bold values indicate statistical significance at the *p* < 0.05 level.

## Data Availability

The datasets used and analysed during the current study are available from the corresponding author upon reasonable request.

## Supplementary Materials

The following supporting information can be downloaded at: https://www.mdpi.com/article/10.3390/ijerph23081050/s1, Figure S1: Noguchi Memorial Institute for Medical Research Review Board and Figure S2: Ghana Health Service Review Committee.

## Abbreviations

AMA	Accra Metropolitan Assembly
DM	Diabetes Mellitus
TB	Tuberculosis

## References

[1] World Health Organization (2024). 2024 Global Tuberculosis (TB) Report.

[2] WHO (2025). Tuberculosis.

[3] WHO (2022). Global Tuberculosis Report [Internet].

[4] Sun H., Saeedi P., Karuranga S., Pinkepank M., Ogurtsova K., Duncan B.B., Stein C., Basit A., Chan J.C.N., Mbanya J.C. (2022). IDF Diabetes Atlas: Global, regional and country-level diabetes prevalence estimates for 2021 and projections for 2045. Diabetes Res. Clin. Pract..

[5] IDF (2025). Diabetes Atlas.

[6] Yorke E., Atiase Y., Akpalu J., Sarfo-Kantanka O., Boima V., Dey I.D. (2017). The Bidirectional Relationship between Tuberculosis and Diabetes. Tuberc. Res. Treat..

[7] Baghaei P., Marjani M., Javanmard P., Tabarsi P., Masjedi M.R. (2013). Diabetes mellitus and tuberculosis: Facts and controversies. J. Diabetes Metab. Disord..

[8] WHO (2011). Collaborative Framework for Care and Control of Tuberculosis and diabetes.

[9] Restrepo B.I. (2016). Diabetes and tuberculosis. Microbiol. Spectr..

[10] Harries A.D., Lin Y., Kumar A.M.V., Satyanarayana S., Takarinda K.C., Dlodlo R.A., Zachariah R., Olliaro P. (2018). What can National TB Control Programmes in low- and middle-income countries do to end tuberculosis by 2030?. F1000Research.

[11] Chang S.H., Cataldo J.K. (2014). A systematic review of global cultural variations in knowledge, attitudes and health responses to tuberculosis stigma. Int. J. Tuberc. Lung Dis..

[12] Huq K.A.T.M.E., Moriyama M., Krause D., Shirin H., Awoonor-Willaims J.K., Rahman M., Rahman M.M. (2022). Perceptions, Attitudes, Experiences and Opinions of Tuberculosis Associated Stigma: A Qualitative Study of the Perspectives among the Bolgatanga Municipality People of Ghana. Int. J. Environ. Res. Public Health.

[13] Mohammedhussein M., Hajure M., Shifa J.E., Hassen T.A. (2020). Perceived stigma among patient with pulmonary tuberculosis at public health facilities in southwest Ethiopia: A cross-sectional study. PLoS ONE.

[14] Tolossa D., Medhin G., Legesse M. (2014). Community knowledge, attitude, and practices towards tuberculosis in Shinile town, Somali regional state, eastern Ethiopia: A cross-sectional study. BMC Public Health.

[15] Sagili K.D., Satyanarayana S., Chadha S.S. (2016). Is Knowledge Regarding Tuberculosis Associated with Stigmatising and Discriminating Attitudes of General Population towards Tuberculosis Patients? Findings from a Community Based Survey in 30 Districts of India. PLoS ONE.

[16] Cremers A.L., De Laat M.M., Kapata N., Gerrets R., Klipstein-Grobusch K., Grobusch M.P. (2015). Assessing the consequences of stigma for tuberculosis patients in urban Zambia. PLoS ONE.

[17] Juma B.M., Mativo Nzioki J., Kibiti C., Shaikh M. (2017). Socio-Cultural Factors Associated with Patient Delay in Commencement of Anti-TB Drugs among TB Patients in Kwale County, Kenya. Afr. J. Health Sci..

[18] Chandru B.A., Varma R.P. (2023). Factors affecting ability of TB patients to follow treatment guidelines—Applying a capability approach. Int. J. Equity Health.

[19] Wieland M.L., Nigon J.A., Weis J.A., Espinda-Brandt L., Beck D., Sia I.G. (2019). Sustainability of a Tuberculosis Screening Program at an Adult Education Center through Community-Based Participatory Research. J. Public Health Manag. Pract..

[20] Retnakumar C., George L.S. (2022). Qualitative Assessment Of The Social Stigma And Discrimination Faced By Tuberculosis Patients Residing In Ernakulam District, Kerala. Int. J. Community Serv..

[21] Ghana Statistical Service (2021). Ghana 2021 Population and Housing Census General Report Volume 3A.

[22] Daniel W.W. (1999). Biostatistics: A Foundation for Analysis in the Health Services.

[23] Datiko D.G., Habte D., Jerene D., Suarez P. (2019). Knowledge, attitudes, and practices related to TB among the general population of Ethiopia: Findings from a national cross-sectional survey. PLoS ONE.

[24] Islam Q.S., Islam M.A., Islam S., Ahmed S.M. (2015). Prevention and control of tuberculosis in workplaces: How knowledgeable are the workers in Bangladesh?. BMC Public Health.

[25] Hossain S., Zaman K., Quaiyum A., Banu S., Husain A., Islam A., Borgdorff M., van Leth F. (2015). Factors associated with poor knowledge among adults on tuberculosis in Bangladesh: Results from a nationwide survey. J. Health Popul. Nutr..

[26] Kwarteng S.O., Donkor E.S., Nweze J.E. (2024). Knowledge, attitude and practices related to tuberculosis among patients at the Presbyterian Hospital in the Asante Akim North District. Afr. Health Sci..

[27] Thapa K., Sharma D., Karki D., Sharma D., Gurung F., Tanwar J., Pariyar L. (2017). Family Health Exercise: Follow-up of an Extra-pulmonary Tuberculosis Patient. J. Gandaki Med. Coll..

[28] Mitku A.A., Dessie Z.G., Muluneh E.K., Workie D.L. (2016). Prevalence and associated factors of TB/HIV co-infection among HIV infected patients in Amhara region, Ethiopia. Afr. Health Sci..

[29] Ugwu K.O., Agbo M.C., Ezeonu I.M. (2021). Prevalence of tuberculosis, drug-resistant tuberculosis and hiv/tb co-infection in Enugu, Nigeria. Afr. J. Infect. Dis..

[30] Alor S.K., Kretchy I.A., Glozah F.N., Adongo P.B. (2024). Community beliefs and practices about diabetes and their implications for the prevention and management of diabetes in Southeast Ghana. BMC Public Health.

[31] Nketiah-Amponsah E., Ahimah-Agyakwah S., Alhassan R.K., Sunkwa-Mills G., Gómez-Pérez G.P., van Andel J., Attachey A.Y.I., Opoku-Boateng Y.N., Addo-Cobbiah V., Okoe-Boye B. (2025). Determinants of lost-to-follow-up (LTFU) among National Health Insurance Scheme-insured hypertension and diabetes patients attending accredited health facilities in Ghana. Trop. Med. Health.

[32] Bosu W.K., Bosu D.K. (2021). Prevalence, awareness and control of hypertension in Ghana: A systematic review and meta-analysis. PLoS ONE.

[33] Taylor L.K., Nyakotey D.A., Kwarteng A. (2023). Physical inactivity and barriers to physical activity among Type-2 diabetics in Kumasi, Ghana. Afr. Health Sci..

[34] Baral S.C., Karki D.K., Newell J.N. (2007). Causes of stigma and discrimination associated with tuberculosis in Nepal: A qualitative study. BMC Public Health.

[35] Jaggarajamma K., Ramachandran R., Charles N., Chandrasekaran V., Muniyandi M., Ganapathy S. (2008). Psycho-social dysfunction: Perceived and enacted stigma among tuberculosis patients registered under revised national tuberculosis control programme. Indian J. Tuberc..

[36] Atre S.R., Kudale A.M., Morankar S.N., Rangan S.G., Weiss M.G. (2004). Cultural concepts of tuberculosis and gender among the general population without tuberculosis in rural Maharashtra, India. Trop. Med. Int. Health.

[37] Zhang Y., Xiao C.M., Zhang Y., Chen Q., Zhang X.Q., Li X.F., Shao R.Y., Gao Y.M. (2021). Factors Associated with Gestational Diabetes Mellitus: A Meta-Analysis. J. Diabetes Res..

[38] Courtwright A., Turner A.N. (2010). Tuberculosis and stigmatization: Pathways and interventions. Public Health Rep..

[39] Sermrittirong S., Van Brakel W.H., Kraipui N., Traithip S., Bunders-Aelen J.F.G. (2015). Comparing the perception of community members towards leprosy and tuberculosis stigmatization. Lepr. Rev..

[40] Chowdhury M.R.K., Rahman M.S., Mondal M.N.I., Sayem A., Billah B. (2015). Social impact of stigma regarding tuberculosis hindering adherence to treatment: A cross sectional study involving tuberculosis patients in Rajshahi City, Bangladesh. Jpn. J. Infect. Dis..

[41] Dodor E. (2008). Health Professionals Expose TB Patients to Stigmatization in Society: Insights from Communities in an Urban District in Ghana. Ghana Med. J..

[42] Chee J., Wong M., Yan J., Chua X., Yi P., Shefaly C. (2024). Effectiveness of educational interventions in reducing the stigma of healthcare professionals and healthcare students towards mental illness: A systematic review and meta-analysis. J. Adv. Nurs..

[43] Sima B.T., Belachew T., Abebe F. (2017). Knowledge, attitude and perceived stigma towards tuberculosis among pastoralists; Do they differ from sedentary communities? A comparative cross-sectional study. PLoS ONE.

[44] Adejumo O.A., Jinabhai C., Daniel O., Haffejee F. (2025). The effects of stigma and social support on the health-related quality of life of people with drug resistance tuberculosis in Lagos, Nigeria. Qual. Life Res..

[45] Amo-Adjei J. (2016). Individual, household and community level factors associated with keeping tuberculosis status secret in Ghana. BMC Public Health.

[46] Ashaba C., Musoke D., Wafula S.T., Konde-Lule J. (2021). Stigma among tuberculosis patients and associated factors in urban slum populations in Uganda. Afr. Health Sci..

[47] Baskaran L., Vasudevan K. (2023). Anandaraj Prevalence of Stigma Among TB Patients and Its Associated Factors-A Community Based Cross-Sectional Study in Puducherry, India. Natl. J. Community Med..

[48] Craig G.M., Daftary A., Engel N., O’Driscoll S., Ioannaki A. (2017). Tuberculosis stigma as a social determinant of health: A systematic mapping review of research in low incidence countries. Int. J. Infect. Dis..

[49] DeSanto D., Velen K., Lessells R., Makgopa S., Gumede D., Fielding K., Grant A.D., Charalambous S., Chetty-Makkan C.M. (2023). A qualitative exploration into the presence of TB stigmatization across three districts in South Africa. BMC Public Health.

[50] Rathnayake R.M.U.K., Rathnayake T.L., Narasinghe U.S., Ranmuthuge R.T.S., Kumari J.U., Rathnayake R.M.J.B. (2020). Prevalence of stigma among pulmonary Tuberculosis patients. J. Postgrad. Inst. Med..

[51] Mohd Salleh S.F., Rahman N.A.A., Rahman N.I.A., Haque M. (2018). Knowledge, attitude and practice towards tuberculosis among community of Kulim Municipal Council, Kedah, Malaysia. Int. Med. J..

[52] Bisallah C.I., Rampal L., Lye M.S., Sidik S.M., Ibrahim N., Iliyasu Z., Onyilo M.O. (2018). Effectiveness of health education intervention in improving knowledge, attitude, and practices regarding Tuberculosis among HIV patients in General Hospital Minna, Nigeria—A randomized control trial. PLoS ONE.

[53] Idris N.A., Zakaria R., Muhamad R., Husain N.R.N., Ishak A., Wan Mohammad W.M.Z. (2020). The effectiveness of tuberculosis education programme in kelantan, malaysia on knowledge, attitude, practice and stigma towards tuberculosis among adolescents. Malays. J. Med. Sci..

[54] Kigozi N.G., Heunis J.C., Engelbrecht M.C., Janse Van Rensburg A.P., Van Rensburg H.C.J.D. (2017). Tuberculosis knowledge, attitudes and practices of patients at primary health care facilities in a South African metropolitan: Research towards improved health education. BMC Public Health.

[55] Speight J., Holmes-Truscott E., Garza M., Scibilia R., Wagner S., Kato A., Pedrero V., Deschênes S., Guzman S.J., Joiner K.L. (2024). Bringing an end to diabetes stigma and discrimination: An international consensus statement on evidence and recommendations. Lancet Diabetes Endocrinol..

[56] Kleinman A. (1980). Patients and Healers in the Context of Culture: An Exploration of the Borderland between Anthropology, Medicine, and Psychiatry.

[57] Fana T.E., Ijeoma E., Sotana L. (2019). Knowledge, Attitudes, and Prevention Practices of Drug Resistant Tuberculosis in the Eastern Cape Province, South Africa. Tuberc. Res. Treat..

[58] Moya E.M., Lusk M.W. (2013). Tuberculosis stigma and perceptions in the US-Mexico border. Salud Publica Mex..

[59] Vaz M., Travasso S.M., Vaz M. (2016). Perceptions of stigma among medical and nursing students and tuberculosis. Indian J. Ethics.

[60] Vericat-Ferrer M., Ayala A., Ncogo P., Eyene-Acuresila J., García B., Benito A., Romay-Barja M. (2022). Knowledge, Attitudes, and Stigma: The Perceptions of Tuberculosis in Equatorial Guinea. Int. J. Environ. Res. Public Health.

