# Supplementary material for: Tuberculosis- and Diabetes Mellitus-Associated Knowledge, Stigma, and Health-Seeking Behaviour in Ghana
Source: Int J Environ Res Public Health. 2026 Aug 13;23(8):1050. doi: 10.3390/ijerph23081050 (PMC13512507; doi:10.3390/ijerph23081050)
Supplement: Supplementary file 1 [file ijerph-23-01050-s001.zip › ijerph-4237629-supplementary.pdf]

UNIVERSITY OF GHANA

NOGUCHI MEMORIAL INSTITUTE FOR MEDICAL RESEARCH (NMIMR)
COLLEGE OF HEALTH SCIENCES
INSTITUTIONAL REVIEW BOARD

13th March 2024

ETHICAL CLEARANCE

FEDERALWIDE ASSURANCE FWA 00001824 **IRB 00001276**

NMIMR-IRB CPN 077/23-24 **IORG 0000908**

On 13th March 2024, the Noguchi Memorial Institute for Medical Research (NMIMR) Institutional Review Board (IRB) conducted a full board meeting review and approved your protocol titled:

**TITLE OF PROTOCOL**	:	**Pan- African Network For Genomic Surveillance Of Poverty Related Diseases and Emerging Pathogens (PANGenS)**
**PRINCIPAL INVESTIGATOR**	:	**Prof Dorothy Yeboah-Manu, PhD.**
**CO-PI(s)**	:	**Profs. Stefan Niemann &Sebastien Gagneux, Drs. Anita Ghansah, Bright Adu, Adwoa Asante-Poku Issac Darko Otchere & Ivy Asantewaa Asante.**

Please note that a final review report must be submitted to the Board at the completion of the study. Your research records may be audited at any time during or after the implementation.

Any modification of this research project must be submitted to the IRB for review and approval prior to implementation.

Please report all serious adverse events related to this study to NMIMR-IRB within seven days verbally and fourteen days in writing.

This certificate is valid till 12th March 2025. You are to submit annual reports for continuing review.

Signature of Chair: ....
Dr. Abraham Hodgson
(NMIMR – IRB CHAIR)

P. O. Box LG 581, Legon, Accra, Ghana | Tel: +233 (0) 302-2916438
Email: nirb@noguchi.ug.edu.gh | www.noguchimedres.org | www.ug.edu.gh

UG

GHANA HEALTH SERVICE ETHICS REVIEW COMMITTEE

In case of reply the number and date of this *Letter should be quoted.*

Research & Development Division
Ghana Health Service
P. O. Box MB 190
Accra
Digital Address: GA-050-3303
Mob: +233-50-3539896
Tel: +233-302-960628
Email: ethics.research@ghs.gov.gh
22nd July 2024

My Ref. GHS/RDD/ERC/Admin/App/24/379
Your Ref. No.

Prof. Dorothy Yeboah-Manu
Noguchi Memorial Institute for Medical Research
University of Ghana
P. O. Box LG 581
Legon - Accra

The Ghana Health Service Ethics Review Committee has reviewed and given approval for the implementation of your Study Protocol.

GHS-ERC Number	**GHS-ERC: 025/03/24**
Study Title	Pan-Africa Network for Genomic Surveillance of Poverty Related Diseases and Emerging Pathogens (PANGenS)
Approval Date	22nd July 2024
Expiry Date	21st July 2025
GHS-ERC Decision	**Approved**

This approval requires the following from the Principal Investigator

- Submission of a yearly progress report of the study to the Ethics Review Committee (ERC)
- Renewal of ethical approval if the study lasts for more than 12 months,
- Reporting of all serious adverse events related to this study to the ERC within three days verbally and seven days in writing.
- Submission of a final report after completion of the study
- Informing ERC if study cannot be implemented or is discontinued and reasons why.
- Informing the ERC and your sponsor (where applicable) before any publication of the research findings.

You are kindly advised to adhere to the national guidelines or protocols on the prevention of COVID -19

Please note that any modification of the study without ERC approval of the amendment is invalid.

The ERC may observe or cause to be observed procedures and records of the study during and after implementation.

Kindly quote the protocol identification number in all future correspondence in relation to this approved protocol

SIGNED...
Mr. Kofi Wellington
(GHS ERC Chairperson)

Cc: The Director, Research & Development Division, Ghana Health Service, Accra
